# Three‐Dimensional Analysis of Occlusal Plane Changes After Clear Aligner Therapy: A Retrospective Study

**DOI:** 10.1155/ijod/8893287

**Published:** 2025-12-29

**Authors:** Domenico Ciavarella, Carlotta Fanelli, Mauro Lorusso, Carmela Suriano, Beatrice Iachini, Michele Laurenziello, Laura Guida, Lucio Lo Russo, Nicola Sgaramella, Rosa Esposito, Michele Tepedino

**Affiliations:** ^1^ Department of Clinical and Experimental Medicine, Dental School of Foggia, University of Foggia, Via Rovelli 50, Foggia, 71122, Italy, unifg.it; ^2^ Mater Dei Hospital, Bari, Italy, deputyprimeminister.gov.mt; ^3^ Department of Biotechnological and Applied Clinical Sciences, Dental School of L’Aquila, University of L’Aquila, Edificio Delta 6 Via Lorenzo Natali, Coppito, 67100, Italy, univaq.it

## Abstract

**Objective:**

The purpose of the present study was to evaluate: (i) occlusal plane (OP) changes after clear aligner therapy (CAT) and (ii) whether such changes correlate with the patient’s facial divergence.

**Materials and Methods:**

Three‐dimensional models of 118 patients (49 males and 59 females; mean ± standard deviation age 22.3 ± 3.4 years) treated with clear aligners were analysed retrospectively. Using CAD‐CAM software, it was possible to define three planes: the reference palatine plane (PP), the OP and the reference vertical maxillary plane. The PP and OP meet mesially, identifying an angle (*α*) in the sagittal view. The intersection of the occlusal and vertical maxillary planes creates two angles in the frontal view: an angle (*β*) for the right side and an angle (*γ*) for the left side. Using Autodesk Fusion 360, the angles were investigated before treatment (T0) and after treatment (T1) to calculate the change for each angle *Δ*(T1–T0). Subsequently, lateral cephalograms were analysed and five measurements were made.

**Results:**

One‐way analysis of variance (ANOVA), showed a significant difference between hyperdivergent and hypodivergent patients for *Δα* and *Δβ*. The post hoc Tukey test showed the following differences: *Δα* was 3.81° greater in hyperdivergent patients compared with hypodivergent patients, and *Δβ* was 1.69° greater in hyperdivergent patients compared with hypodivergent patients.

**Conclusion:**

Treatment with aligners did not lead to a clinically significant change in the OP. However, when dividing the sample into groups based on craniofacial divergence, OP orientation change differed significantly between the three groups.

**Clinical Significance:**

According to the present study, aligners may be a useful therapeutic option for hyperdivergent patients and a less favourable option for hypodivergent patients and patients with occlusal asymmetries, both structural and functional, who may need particular treatment approaches and diagnostic evaluations.

## 1. Introduction

Occlusal plane (OP) orientation is an important factor to determinate correct occlusion in orthodontic treatment [1, 2]. This parameter is connected with stomatognathic function and aesthetic dentofacial demands [3, 4]. The direction of chewing forces and masticatory movement is altered by clockwise or counterclockwise rotation of the OP, potentially acting as an adaptive response to the temporomandibular joint (TMJ) [5–7]. Due to the ideal correlation between the curvature of the maxillary incisal and canine edges and the curvature of the lower lip, OP modifications could affect the attractiveness of the smile arc [4]. Researchers have investigated the effects of traditional multibrackets and functional orthodontic treatment on OP orientation. OP orientation is determined especially by the vertical position of the maxillary teeth and the extrusion and intrusion of the molars and premolars [8]. According to these aspects, OP changes during orthodontic treatment are largely related to vertical control of the maxillary and mandibular teeth [9, 10].

Orthodontists are using clear aligner therapy (CAT) to satisfy adult patients’ aesthetic requirements. Researchers have mainly examined the predictability of successful treatment, favourable periodontal features, decreased risk of apical resorption and spot lesions [11–15]. Despite the widespread use of CAT, there is a lack of studies specifically addressing changes in OP orientation in relation to skeletal divergence, particularly in the absence of prescribed intrusive or extrusive movements. To date, only the pilot study by Ciavarella et al. [16] has suggested a tendency for counterclockwise rotation of the OP in hyperdivergent patients and clockwise rotation in hypodivergent patients. Another study focusing on the curve of Spee reported greater intrusion of the second molars in hypodivergent subjects compared to hyperdivergent ones. However, none of these studies assessed changes in the OP inclination in the frontal view (occlusal cant), which is an innovative focus of the present investigation [17].

The aim of the present study was to evaluate changes in the OP during CAT in patients with upper and lower crowding, within a treatment plan that did not involve extrusion or intrusion of the posterior teeth. As a secondary outcome, the changes in the OP were compared among different skeletal divergence patterns. The null hypotheses are (1) there is no difference between pre‐ and post‐treatment OP orientation and (2) there is no difference in OP changes among the different facial divergence patterns.

## 2. Materials and Methods

This retrospective study was designed following the Strengthening the Report of Observational Studies in Epidemiology (STROBE) guidelines for observational studies [18]. The procedures followed the Declaration of Helsinki and were approved by the Ethics Committee of the University. A total of 118 Caucasian patients (49 males and 59 females; mean ± standard deviation age 22.3 ± 3.4 years) were treated at the Department of Orthodontics, University of XXX, Italy. All patients signed a written informed consent form to participate and were based on the inclusion and exclusion criteria listed in Table 1. The treatment setup aimed to resolve moderate crowding, avoiding extrusion or intrusion of the molars and incisors. The average treatment time was 6 ± 1 months. Horizontal attachments were placed on posterior teeth to improve aligner fitting. Moderate crowding was solved through interproximal enamel reduction (IPR). Little’s index [19], which calculates the separation between the anterior teeth’s anatomical contact points, was used to evaluate crowding. A power analysis (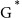
Power 3.1.9.2, Franz Faul, Universitat Kiel, Germany) revealed that to detect a large effect size of 0.4 [20] with a one‐way analysis of variance (ANOVA), an *α* of 0.05 and power (1−*β* error probability) of 0.95, 84 subjects would be needed [20].

**Table 1:** Inclusion and exclusion criteria.

Inclusion criteria	Exclusion criteria
Non growing patients (skeletal age CS6 according to the cervical vertebral maturation method)	Use of clear aligners in conjunction with auxiliaries
Non extraction treatment	Periodontal disease
Clear aligner system (Dooris) with a tickness of 0.75 mm	Implants or root canal therapies before and during the orthodontic treatment
	Individuals with one or more agenesis or impacted teeth
Sequence of 15 aligners or more	Temporomandibular disorders
Moderate crowding (4–6 mm) according to the little index	Destructive caries
	Maxillo‐facial congenital syndrome and Skeletal malformations

A direct scan of the maxillary and mandibular arches was carried out using an intraoral scanner (TRIOS; 3Shape, Copenhagen, Denmark), in accordance with the manufacturer’s suggested protocol, before and after treatment. The Standard Triangle Language (STL) files were imported into dental CAD software (Meshmixer, Autodesk Inc.) to generate virtual models. OP changes in the frontal and sagittal views were analysed. OP changes in the sagittal view were investigated using two planes: the maxillary OP and the reference palatine plane (PP). The first plane passes through the palatal cusps of first premolar and the mesio‐palatal cusp tips of the maxillary first molar; the second plane passes through the bilateral first palatal rugae and the incisive papilla (Figure 1). An angle (*α*) results from the intersection between the two planes. This angle was measured before (T0) and after (T1) CAT by using Autodesk Fusion 360 to obtain the change in *α* (*Δα*, *α* at T1–*α* at T0) in the sagittal view.

Figure 1Digital procedure to evaluate occlusal plane changes in the sagittal view. (a) An occlusal view of the maxillary STL model. (b) Three‐point insertion of the reference palatine plane. (c) The maxillary palatine reference plane and four‐point insertion of the occlusal plane. (d) Sagittal intersection between the two planes to obtain the angle (*α*).

**Figure:**
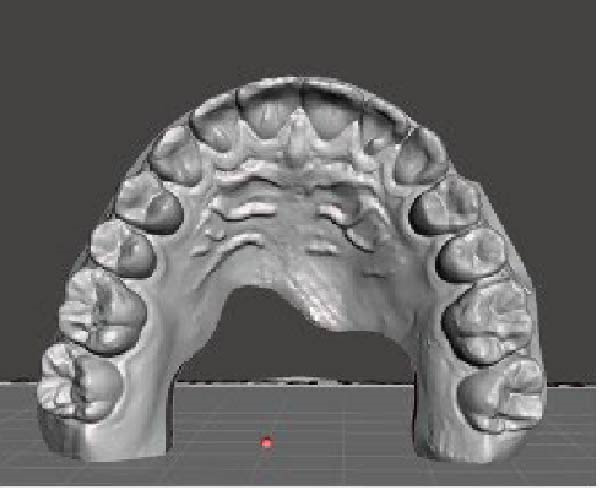
(a)

**Figure:**
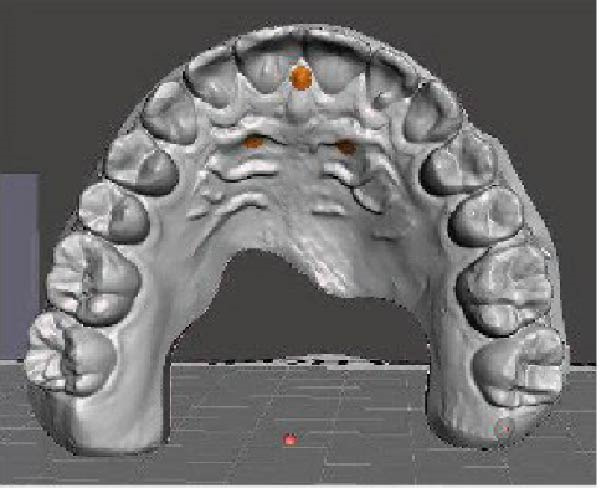
(b)

**Figure:**
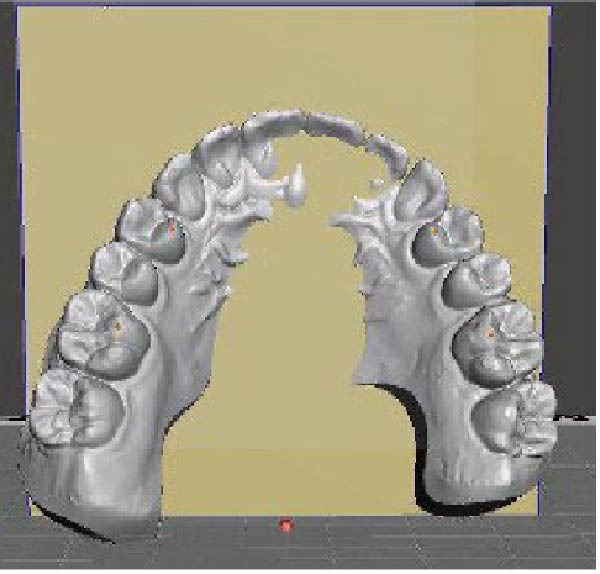
(c)

**Figure:**
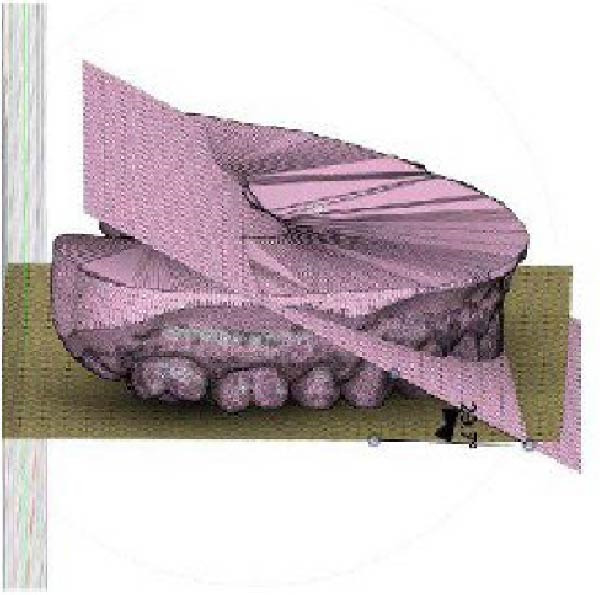
(d)

The frontal view was evaluated based on the same maxillary OP as in the sagittal view, and a second reference vertical PP passing through the median palatine suture was considered. An angle (*β*) results from the intersection between the two planes in the frontal view on the right side of the arches. The same intersection on the left side results in another angle (*γ*). Using Fusion 360, these angles were measured before (T0) and after (T1) CAT to obtain the change in *β* (*Δβ*, *β* at T1 – *β* at T0) and the change in γ (*Δγ*, γ at T1–*γ* T0) in the frontal view (Figure 2).

Figure 2Digital procedure to evaluate occlusal plane changes in the frontal view. (a) An occlusal view of the maxillary STL model and vertical plane passing through the median palatine suture. (b) The intersection between the vertical reference plane and the occlusal plane with four‐point insertion. (c) Frontal intersection between the two planes to obtain the angle (*β*) on the right side. (d) Frontal intersection between the two plane to obtain the angle (*γ*) on the left side.

**Figure:**
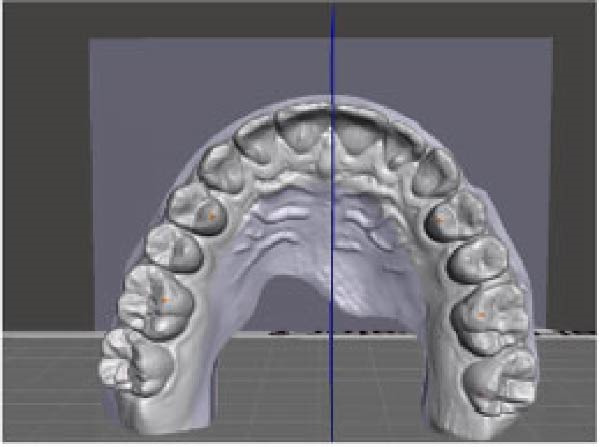
(a)

**Figure:**
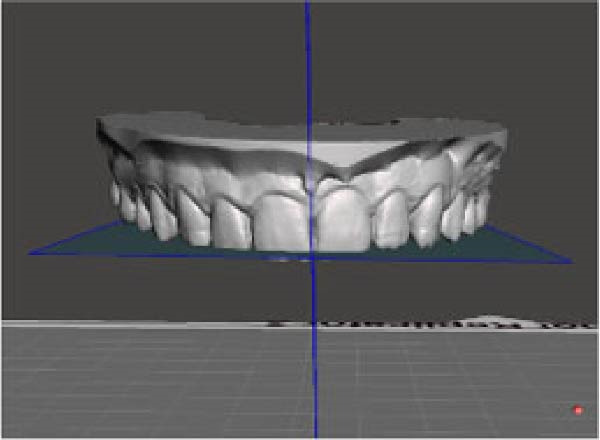
(b)

**Figure:**
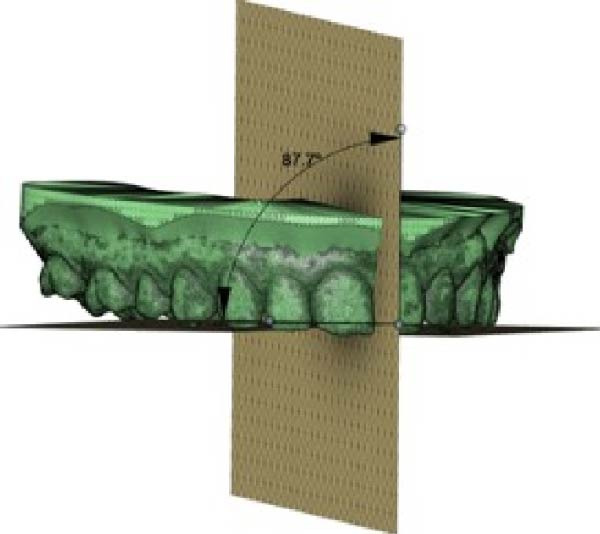
(c)

**Figure:**
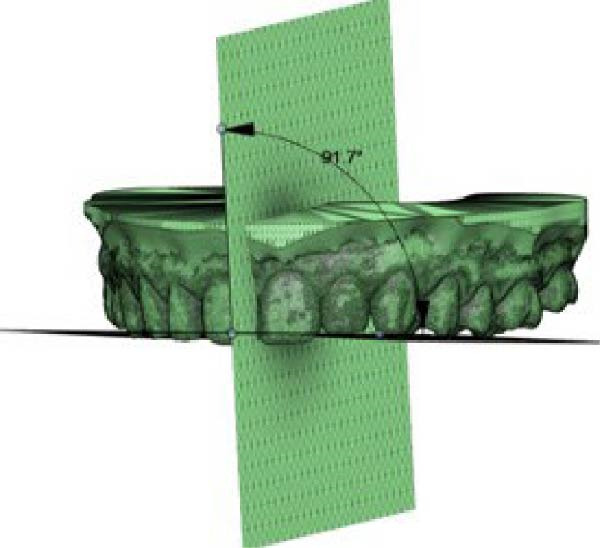
(d)

The dental measurements were performed for each digital scan of the maxillary arches and are described in Table 2.

**Table 2:** List and definition of all the cephalometric measurements used in the present study.

Cephalometric measurement	Definition
PP‐MP	The angle between the palatine plane (PP), passing through Ans and Pns, and mandibular plane (MP)
PP‐OP	The angle between the palatine plane (PP), passing through Ans and Pns, and occlusal Plane
OP‐MP	The angle between the occlusal plane (OP), passing through the incisal edge of U1 to the midpoint of the U6 on the occlusal, and mandibular Plane (MP)
AFH	The anterior facial height (Na‐Me) expressed in percentage
PFH	The posterior facial height (S‐Go)

**OP angular° measurements**	

*Δα* (*α*1–*α*0)	Post‐treatment difference for the angle between the occlusal plane and the occlusal palatine reference plane
*Δβ* (*β*1–*β*0)	Post‐treatment difference for the angle between the occlusal plane and maxillary transverse plane on the right side
*Δγ* (*γ*1–*γ*0)	Post‐treatment difference for the angle between the occlusal plane and maxillary transverse plane on the left side

Prior to treatment, lateral head cephalograms were obtained for each patient. The sample was divided into three groups according to the SN‐MP angle [21]:

• Group 1—SN‐MP > 35.5° (hyperdivergent patients);
• Group 2—30.5° ≤ SN‐MP ≤ 35.5° (normodivergent patients);
• Group 3—SN‐MP < 30.5° (hypodivergent patients)

Lateral radiographs were analysed using the FastCeph software (Caes Software, Grottaferrata, Italy). To minimise measurement errors, cephalometric radiographs and models analysis were selected randomly and reanalysed 20 days later by the same trained examiner. The following cephalometric variables, described in Table 2, were analysed: PP‐MP, PP‐OP, OP‐MP, AFH and PFH.

### 2.1. Statistical Analysis

The random error of each measurement was calculated using Dahlberg’s formula (*S* = ∑ *d* ^2^/2*N*), where *d* is the difference between the first and second measurements and *N* is the number of radiographs evaluated [22, 23]. The random error of cephalometric measurements ranged from 0.10 to 0.24 mm for linear measurements and from 0.12° to 0.31° for angular measurements. The random error of OP angular measurements ranged from 0.011° to 0.019°. Data were analysed using GraphPad Prism software 6.0 (GraphPad Prism Software, San Diego, CA, USA). The Shapiro–Wilk test was used to determine whether the data were normally distributed (Table 3). Descriptive statistics were also calculated (Table 4). Differences between the three craniofacial divergence groups in *Δα*, *Δβ* and *Δγ* were assessed using one‐way ANOVA followed by post hoc Tukey test (Tables 5 and 6). A *p*‐value < 0.05 was considered to be statistically significant.

**Table 3:** Results of the KOLMOGOROV–SMIRNOV test for all variables of the whole sample and group.

	Statistics	gf	Sign
*Δα*	0.912	118	0.001
*Δβ*	0.961	118	0.002
*Δγ*	0.986	118	0.183

*Note:* Statistically significant for *p* < 0.05.

**Table 4:** Descriptive statistic of OP modification among the groups.

Cohort	*Δα* sagittal (°)	*Δβ* frontal dx (°)	*Δγ* frontal sx (°)
Normodivergents (*n* = 40)	1.80	0.41	0.07
Hyperdivergents (*n* = 42)	2.88	1.69	−0.34
Hypodivergents (*n* = 36)	−0.95	−0.02	−0.23
Total sample (*n* = 118)	1.16	0.65	−0.16

**Table 5:** One‐way ANOVA test for OP changes measurements between the three groups.

	Sum of squares	dl	Mean square	*F*	Sig.
*Δα*					
Between groups	306.33	2	153.16	4.274	0.016 ^∗^
Within groups	4121.03	115	35.83	—	—
Total	4427.36	117	—	—	—
*Δβ*					
Between groups	59.07	2	29.53	3.580	0.031 ^∗^
Within groups	948.93	115	8.25	—	—
Total	1008.01	117	—	—	—
*Δγ*					
Between groups	3.68	2	1.84	0.220	0.803
Within groups	964.03	115	8.38	—	—
Total	967.71	117	—	—	—

^∗^Statistically significant for *p* < 0.05.

**Table 6:** Tukey’s post hoc test.

Dependent variable	(I) Group	(J) Group	Mean difference (I–J)	Std. error	*p*	95% confidence interval	
						Lower bound	Upper bound
*Δα*	Normodivergent	Hyperdivergent	2.72	1.32	0.102	−0.41	5.86
	Normodivergent	Hypodivergent	−1.08	1.37	0.71	−4.34	2.18
	Hyperdivergent	Hypodivergent	3.81 ^∗^	1.35	0.01	−7.03	−0.58

*Δβ*	Normodivergent	Hyperdivergent	0.41	0.63	0.79	−1.09	1.92
	Normodivergent	Hypodivergent	−1.27	0.65	0.13	−2.84	0.29
	Hyperdivergent	Hypodivergent	1.69 ^∗^	0.65	0.02	−3.24	−0.14

*Δγ*	Normodivergent	Hyperdivergent	0.31	0.63	0.87	−1.20	1.83
	Normodivergent	Hypodivergent	0.41	0.66	0.80	−1.16	1.99
	Hyperdivergent	Hypodivergent	0.10	0.65	0.986	−1.45	1.66

^∗^Statistically significant for *p* < 0.05.

## 3. Results

The Shapiro–Wilk test (Kolmogorov–Smirnov test) showed that *Δα*, *Δβ* and *Δγ* were normally distributed (Table 3). Table 4 presents the OP changes for the entire sample as well divided into the three groups based on divergence for the sagittal and frontal views. For the sagittal view, the entire sample showed a slight modification of the OP (1.1°). After dividing the sample into the three groups based on divergence, there were certain variations. Normodivergent patients showed a small increase in *Δα* (1.8°) despite negligible variations. Hyperdivergent patients exhibited an increase in *Δα* (2.88°) while hypodivergent presented a decrease in *Δα* (−0.95°). For the frontal view, the biggest changes were in the maxillary right side; although the changes were slight, there was an increase in the distance to the reference plane (total sample 0.65°; hyperdivergent: 1.69°; normodivergent: 0.4°). On the other hand, hypodivergent patients presented a negligible reduction in *Δβ* (−0.029°). All groups showed a reduction in *Δγ* (total sample: −0.16°; hyperdivergent: −0.34°; hypodivergent: −0.23°; normodivergent: 0.077°).

When comparing the OP changes between the three groups, one‐way ANOVA (Table 5) showed a significant difference for only *Δα* and *Δβ*. Post hoc testing (Table 6) revealed the following significant differences:

• *Δα* in hyperdivergent group was 3.81° greater than in hypodivergent group;
• *Δβ* in hyperdivergent group was 1.69° greater than in hypodivergent group.

Based on these results, the null hypotheses were partially rejected.

## 4. Discussion

The present study investigated alterations in OP orientation in patients with upper and lower crowding and different vertical patterns who underwent CAT. The treatment approach involved an alignment setup with IPR without expansion. A virtual reference PP was created to measure the angle between the OP on the three‐dimensional models and this plane to evaluate OP changes in the sagittal view. The reference OP was drawn passing through the midpoint of the first palatal rugae and the interincisal papilla. Based on prior studies, these anatomical landmarks are reliable sources and when used, reduce interindividual variability, ensuring consistent data [24, 25]. Similarly, for the frontal view analysis, a reference plane was used that passed through the median palatine suture, a stable anatomical structure in the maxilla of adult patients [26, 27].

In the present study, the mean age of the patients was 22.5 years when the treatment started. Therefore, residual growth and skeletal maturity did not induce changes in OP orientation [28]. Because growth‐related factors such as vertical eruption of teeth and dentoalveolar bone can be excluded, changes in OP orientation during orthodontic treatment are a consequence of mesial molar movement, vertical control of the maxillary and mandibular molars and extrusion and intrusion of the incisors [1, 29]. As OP orientation can be modified by orthodontic treatment, it is an essential element to consider for correct diagnosis and treatment planning to achieve proper occlusion and stable results.

Researchers have investigated the effects of orthodontic multibrackets treatment on OP orientation. Fixed appliance therapy tends to extrude teeth, which increases the mandibular plane (MP) angle [30].

In addition, class II elastics, commonly used to correct class II malocclusion, can cause clockwise rotation of the OP due to mandibular molars and maxillary incisors extrusion [1, 31]. Conversely, class III elastics induce a counterclockwise rotation and can consequently modify the patient’s vertical dimension [1].

Although clear aligners are increasingly used to address simple crowding, few studies have been conducted to evaluate aspects related to the potential unexpected effects of these appliances [17, 32, 33]. The plastic thickness between the arches, along with occlusal forces, may be able to control or influence the intrusion of the posterior teeth. There is evidence of the ‘bite block effect’ based on the frequent recurrence of posterior open bite in patients following CAT [34, 35] and the favourable resolution of open bite [36].

According to the results of the present study, CAT did not cause any clinically or statistically significant alterations in the OP during treatment. However, there were changes in OP orientation following treatment when the patients were grouped according to craniofacial divergence. In hyperdivergent patients, the angle between the reference OP and maxillary PP (*Δα* = 3.81°, *p* < 0.05) increased compared with hypodivergent patients after CAT in the sagittal view. In hypodivergent patients, the shorter distance of the premolar and first molar cusps from the maxillary reference plane indicates an intrusion of these teeth and a clockwise rotation of the OP. Furthermore, there was a significant difference between hyperdivergent and hyperdivergent patients (*Δβ* = 1.69°, *p* < 0.05) on the right side in the frontal view. This suggests that premolars and molars in hypodivergent patients experienced more intrusion on the right side after CAT compared to hyperdivergent patients. No significant differences were found between normodivergent patients and either hypo‐ or hyperdivergent patients in either view.

The data suggest different effects on OP orientation among the three groups. Specifically, in hyperdivergent patients, the OP shows a counterclockwise rotation after CAT, while in hypodivergent patients, there is a clockwise rotation. It could be hypothesised that the modification of occlusal stress induced by aligners leads to immediate muscular changes, with a consequent alteration in tooth position that results in OP changes.

There may be important variations in muscle activity when the quantity and quality of occlusal contacts are altered [37, 38]. Dincer and Aslan [39] reported that the number of occlusal contacts affects the bite force during clenching by up to 20% and that the number of occlusal contacts is related to EMG activity of masticatory muscles. There are limited studies related to EMG activity, occlusal contact and the bite force during CAT. Manfredini et al. [40] found no detectable changes in the EMG activity of an adult patient wearing a thermoplastic retainer [41]. On the contrary, Okeson [42] reported that the masseter and temporalis muscles became more active when wearing soft splints. Moreover, Castroflorio and Tecco [33, 43] observed an increase in muscle activity shortly after aligner therapy, and Sultana [33, 43] showed a significant increase in occlusal contacts following CAT. According to Tepedino et al. [44], when transparent aligners are worn during orthodontic treatment, the centre of force (COF) shifts posteriorly and sagittally. Furthermore, this analysis showed that the COF position in the frontal view did not exhibit any observable asymmetries in the short term.

A potential rationale for OP changes, based on divergence in the sagittal view, could be the intrusion of molars and premolars in hypodivergent patients and greater intrusion of premolars in hyperdivergent patients. Specifically, variations in muscle strength and the occlusal barycentre may be connected to the variable orientation of the OP according to the divergence pattern. Hyperdivergent patients exhibit a lower bite force related to the posterior occlusal barycentre and a clockwise rotation of the mandible and the OP; in contrast, hypodivergent patients have a greater bite force and a related occlusal anterior barycentre [45]. Given that higher bite forces noted while going from incisors to molars, greater intrusion would be expected in patients with stronger muscles, especially in the posterior area of the arch.

Studies have also revealed that hypodivergent patients have more anterior occlusal contacts than hyperdivergent patients [46]. Early anterior contact and the thickness of the aligners can contribute to the loss of posterior contact [47]. Therefore, the control of molar verticality and the potential intrusion of the premolars may be explained by the decreased occlusal force and fewer anterior contacts in hyperdivergent patients. It could be hypothesised that premolars, being smaller and having fewer roots, are more susceptible to intrusion despite the reduction in occlusal force.

In the frontal view, the cant of the posterior OP often reflects the vertical height of occlusion and may be linked to the prevalence of chewing cycles. A shorter vertical height of the dentition during dentoskeletal maturation influences the mandibular position, resulting in a transverse inclination of the OP [48]. Muscular activity may be altered by contralateral changes in the occlusal vertical dimension, which could result in mandibular asymmetry and condylar shift during functional movement [49]. It is possible to hypothesise that the patients in the sample had a mild vertical occlusal height or a primary chewing side on the right. The thickness of the aligners during CAT may have highlighted an initial muscular imbalance, causing alterations in tooth position and a reduction in the vertical dimension on the initially dominant side (right). On the right side in the frontal view, hyperdivergent patients showed a greater increase in *Δb*, implying a greater reduction in the vertical dimension compared to hypodivergent patients. This might be because, while hyperdivergent patients have weaker muscles, their greater initial vertical dimension allows for a larger change. Based on this study, aligners may be a useful therapeutic option for hyperdivergent patients but a less favourable one for hypodivergent patients and those with structural and functional occlusal asymmetries, who might require specific clinical strategies and diagnostic assessments.

### 4.1. Limitation of the Study

First, although selection bias may have been introduced by the retrospective nature of patient enrolment, attempts were made to reduce it by rigorously adhering to consecutive enrolment. Second, the applicability of the results to a wider population is restricted by the limited sample size. Third, the method used for digital measurements requires operators with knowledge of digital programs. Final, the patient selection process did not assess factors related to muscle and masticatory function.

Nonetheless, the study exhibits several significant strengths, including a digitally integrated and repeatable workflow, as well as a robust sample size justified by appropriate power analysis. Moreover, it represents an innovative contribution to the field, as no prior studies have investigated this topic using a comparable sample size in conjunction with a standardised and reproducible methodological approach. Additional studies are necessary to gain a comprehensive understanding of the effects of CAT on the levelling of the OP.

## 5. Conclusion

The present study evaluated the OP after CAT in patients with different craniofacial divergence patterns. CAT did not result in a clinically significant modification of the OP after a mean treatment duration of 13 months in the frontal and sagittal views. In conclusion, after dividing the sample into groups based on craniofacial divergence, the following take home messages were made:

• Hyperdivergent patients showed counterclockwise rotation of the OP compared with hypodivergent patients.
• Hypodivergent patients showed minimal changes in the OP with clockwise rotation compared with hyperdivergent patients.
• Normodivergent patients showed minimal changes in the OP, which were in line with the treatment plan and considered favourable.
• Hyperdivergent patients showed greater frontal inclination of OP on the right side compared with hypodivergent patients.

## Ethics Statement

All the procedures of this research protocol adhered to the Declaration of Helsinki and were approved by the Ethics Committee of the University of L’Aquila (Approval no: 39873/04/2020). Informed consent was obtained from all individual participants included in the study.

## Disclosure

All authors read and approved the final manuscript.

## Conflicts of Interest

The authors declare no conflicts of interest.

## Author Contributions

Domenico Ciavarella is responsible for the treatment planning decision and clinical patient treatment. Carlotta Fanelli and Mauro Lorusso did the article test production. Beatrice Iachini and Michele Laurenziello had a hand in the digital elaboration set‐up and planning. Laura Guida, Carmela Suriano and Lucio Lo Russo led the clinical treatment of the patient. Rosa Esposito is responsible for the production of tables and figures, Nicola Sgaramella and Michele Tepedino contributed in the treatment planning decision and clinical patient treatment.

## Funding

The authors declare that they have not received funding. Open access publishing facilitated by Universita degli Studi di Foggia, as part of the Wiley ‐ CRUI‐CARE agreement.

## Data Availability

The datasets used and/or analysed during the current study are available from the corrisponding author on reasonable request.
